# Supplementary material for: Comparative genomics provides new insights into the diversity, physiology, and sexuality of the only industrially exploited tremellomycete: Phaffia rhodozyma
Source: BMC Genomics. 2016 Nov 9;17:901. doi: 10.1186/s12864-016-3244-7 (PMC5103461; doi:10.1186/s12864-016-3244-7)
Supplement: Additional file 6: — List of orphan genes with links to PFAM (related to Additional file 1: Table S1). (ZIP 1428 kb) [file 12864_2016_3244_MOESM6_ESM.zip › PFAM_UNIQUE/G02779_P.html]

#                                                                            --- full sequence --- -------------- this domain -------------   hmm coord   ali coord   env coord
  
# target name        accession   tlen query name           accession   qlen   E-value  score  bias   #  of  c-Evalue  i-Evalue  score  bias  from    to  from    to  from    to  acc description of target
  
#------------------- ---------- ----- -------------------- ---------- ----- --------- ------ ----- --- --- --------- --------- ------ ----- ----- ----- ----- ----- ----- ----- ---- ---------------------
  
G02779\_P             -            741 bZIP\_1               PF00170.16    64   5.5e-05   21.7   6.6   1   1   5.7e-07   0.00014   20.5   6.6     6    46   460   500   456   509 0.92 - bZIP transcription factor  
G02779\_P             -            741 bZIP\_2               PF07716.10    54   0.00028   19.3   7.2   1   1   3.2e-06   0.00092   17.7   7.2     3    41   457   496   455   500 0.93 - Basic region leucine zipper  
G02779\_P             -            741 DUF3035              PF11233.3    157      0.26   10.0   2.5   1   2   0.00019      0.58    8.9   0.9    35   117   316   398   311   431 0.65 - Protein of unknown function (DUF3035)  
G02779\_P             -            741 DUF3035              PF11233.3    157      0.26   10.0   2.5   2   2       0.5   1.5e+03   -2.2   0.0    55    82   693   719   686   721 0.71 - Protein of unknown function (DUF3035)  
G02779\_P             -            741 GN3L\_Grn1            PF08701.6     80    0.0074   14.8   1.0   1   3      0.61   1.2e+03   -1.9   2.2    13    31   458   476   449   483 0.48 - GNL3L/Grn1 putative GTPase  
G02779\_P             -            741 GN3L\_Grn1            PF08701.6     80    0.0074   14.8   1.0   2   3   3.7e-06    0.0074   14.8   1.0    34    67   535   568   525   581 0.85 - GNL3L/Grn1 putative GTPase  
G02779\_P             -            741 GN3L\_Grn1            PF08701.6     80    0.0074   14.8   1.0   3   3         3     6e+03   -4.7   1.1    53    64   641   652   635   663 0.43 - GNL3L/Grn1 putative GTPase
